# CBCT Assessment of Root Dentine Removal by Gates-Glidden Drills and Two Engine-Driven Root Preparation Systems

**DOI:** 10.22037/iej.2017.06

**Published:** 2017

**Authors:** Azade Harandi, Fatemeh Mohammadpour Maleki, Ehsan Moudi, Maryam Ehsani, Soraya Khafri

**Affiliations:** a *Dental Material Research Center, Department of Endodontics, Dental School, Babol University of Medical Sciences, Babol, Iran; *; b *Student Research Committee, Dental School, Babol University of Medical Sciences, Babol, Iran; *; c *Dental Material Research Center, Department of Radiology, Dental School, Babol University of Medical Sciences, Babol, Iran; *; d *Department of Social Medicine and Health, Dental School, Babol University of Medical Sciences, Babol, Iran*

**Keywords:** Cone-Beam Computed Tomography, Maxillary First Premolar, Root Canal Preparation, Root Thickness

## Abstract

**Introduction:**

The aim of this study was to compare the dentine removing efficacy of Gates-Glidden drills with hand files, ProTaper and OneShape single-instrument system using cone-beam computed tomography (CBCT).

**Methods and Materials:**

A total of 39 extracted bifurcated maxillary first premolars were divided into 3 groups (*n*=13) and were prepared using either Gates-Glidden drills and hand instruments, ProTaper and OneShape systems. Pre- and post-instrumentation CBCT images were obtained. The dentin thickness of canals was measured at furcation, and 1 and 2 mm from the furcation area in buccal, palatal, mesial and distal walls. Data were analyzed using one-way ANOVA test. Tukey’s post hoc tests were used for two-by-two comparisons.

**Results:**

Gates-Glidden drills with hand files removed significantly more (*P*<0.001) dentine than the engine-driven systems in all canal walls (buccal, palatal, mesial and distal). There were no significant differences between OneShape and ProTaper rotary systems (*P*>0.05).

**Conclusion:**

The total cervical dentine removal during canal instrumentation was significantly less with engine-driven file systems compared to Gates-Glidden drills. There were no significant differences between residual dentine thicknesses left between the various canal walls.

## Introduction

The success of endodontic treatment depends on the perfect access to the pulp chamber and root canals, cleaning and shaping and perfect obturation of the root canal system [1, 2]. Preparation of the cervical and middle third of root canals is an important step for improving the definition of anatomical diameter at working length [3] and gives the dentist better control of the files in the apical third [4-7], decreasing the possibility of ledge formation, apical transportation, perforation and file fracture [8]. Enlargement of the cervical third facilitates cleaning and shaping, irrigation and three-dimensional filling of the root canals [7]. Gates-Glidden (GG) drills are the first instruments used to enlarge the cervical portion of root canals [9, 10]. They are commonly used during endodontic procedures for their ease of use and low cost [8, 9]. Many studies have been conducted that resulted in improving the raw materials of rotary instruments to improve their clinical efficiency [7, 11-13]. Nickel-Titanium (Ni-Ti) engine-driven instruments allow more centered and better tapered preparation of the root canals, in addition they are easier and faster than stainless steel instruments [11, 14]. ProTaper system (Dentsply Maillefer, Ballaigues, Switzerland) is one of the widely used rotary systems that is specially characterized by progressive taper, and convex, triangular cross-section design, with a positive rake angle, a modified guiding tip, different helical angle and balanced pitches [2]. Recently, single-file concept that facilitates and fastens root canal preparation has gained interest [15-17]. The single-file NiTi systems such as WaveOne (Dentsply, Maillefer, Ballaigues, Switzerland), Reciproc (VDW, Munich, Germany), and OneShape (MicroMega, Besancon, France) apply only one instrument [2, 18]. 

OneShape is one of the most recently introduced rotary NiTi single file systems [2, 18]. It has been used in continuous rotary motion in contrast to the other recently introduced single file systems which apply reciprocal back and forth motion [2, 18, 19]. This single file is available in one size (#25/0,06) and different lengths (21, 25 and 29 mm) which is used at a speed of 350-450 rpm and a torque of 4.5 N with a pecking motion [2, 18]. This rotary file has different cross-sectional designs that change from 3 to 2 cutting edges between the apical and coronal thirds [2, 14, 18-20]. 

Fracture of endodontically treated teeth increases proportionally with an increase in the amount of root dentine removal [21, 22], especially in maxillary and mandibular premolars, due to their narrow mesiodistal width [23-25]. One of the unique anatomical features of maxillary premolars is the presence of deep mesial root concavities [26-28], that increase fracture susceptibility for lower dentine thickness [23, 27]. The palatal groove of the buccal root is another characteristic in the bifurcated maxillary first premolars. The prevalence of this landmark ranges from 62 to 100% [26, 28, 29]. This anatomical feature may present endodontic difficulties such as perforation of the dentineal wall during preparation of the root canals [26, 28, 30]. 

Cone-beam computed tomography (CBCT) is a noninvasive three-dimensional (3D) imaging technique [31-33] which has been used in root canal therapy for the assessment of root canal preparation, obturation and retreatment [11, 33]. It can determine the amount of dentine removed during preparation of root canals by measuring the dentine thickness before and after instrumentation [20]. Many studies have compared the amount of root dentine removal in manual files and rotary instruments [34-36] and also various types of rotary instruments [12-15]. 

The aim of this *in vitro* study was comparative assessment of root dentine thickness after canal preparation with Gates-Glidden drills and hand files, ProTaper and OneShape instruments, in bifurcated maxillary first premolars using cone-beam computed tomography (CBCT).

## Materials and Methods

In this experimental study, a total of 39 human bifurcated maxillary first premolars with mature apices were selected from a pool of recently extracted teeth. The teeth were disinfected in 5% NaOCl solution for 24 h and debrided of periodontal tissues and calculus with an ultrasonic scaler (EMS Piezon Master 400, CH-1260 Nyon, Switzerland). The bifurcation was not located more apically than the coronal third of roots. In all teeth, the palatal groove of the buccal root originated from the bifurcation. Teeth with internal or external root resorption, fracture and calcified root canals were excluded from this study. 

The cusps of all the teeth were flattened to have a stable reference point. Standard access cavities were prepared. Two periapical radiographs of each tooth with a #10 K-file (Mani, Tochigi, Japan) inserted in each canal were taken from the mesial and buccal aspects; then only the teeth with a moderate root canal curvature (10 to 35 degrees according to Schneider’s method [37]), were included. The working lengths of the canals was obtained by observing the tip of the file protruding through the apical foramen and subtracting 1 mm from the recorded length. The teeth were mounted on quadrangular models embedded in a type III gypsum cast (Mold Stone, Dental Pars Co, Iran) and positioned for primary scans of CBCT before preparation. The specimens were identically positioned in a special device with no changes in mesiodistal and buccolingual orientation, which allowed pre- and postoperative images to be compared. The scans were obtained using a Newtom 5G CBCT unit (Quantitative Radiology SRL Co., Verona, Italy) with a scan time of 20 sec, 75 kVp, 8 mA and a voxel size of 8×8 mm. Then 1 mm axial cross-section, 3 cut plans at furcation and below furcation (1 and 2 mm), were obtained by NTT Viewer software program (NTT Software Corporation, Yokohama, Japan). With NTT software program, dentine thickness was measured at a distance from the canal walls perpendicular to the external surface of the root. This technique was used for the mesial, distal, lingual and buccal walls of each section of buccal and lingual roots. Data were saved for comparison with postoperative scans. Before the preparation, #10 and #15 K-files (Mani, Tochigi, Japan) were inserted into the root canal up to the working length for checking the canal patency. 

Then the teeth were randomly assigned to 3 groups as follows: Group I (*n*=13): The samples were prepared with #1, 2 and 3 GG drills (Dentsply, Maillefer, Ballaigues, Switzerland) at 1200 rpm. The drills were used with straight up-and-down motions. Then the step-back technique was conducted with hand K-files (Dentsply, Maillefer, Ballaigues, Switzerland) for cleaning and shaping. Canal preparation was accomplished till the master apical file size (#25) was reached. In group II (*n*=13), preparation was carried out with SX, S1, S2, F1 and F2 ProTaper rotary files (Dentsply, Maillefer, Ballaigues, Switzerland) system. Canal preparation was completed in a single-length technique according to manufacturer’s instructions. Finally in group III (*n*=13), the cervical third of each root canal was prepared with Endoflare files (Micro Mega, Besancon, France). After pre-flaring, OneShape file (25/0.06) (Micro Mega, Besancon, France) was used for cleaning and shaping according to the manufacturer’s recommendations.

In all groups, pre-flaring was done 3 mm bellow the furcation. The rotary instruments were installed on an electric motor (Endo-Mate TC, NSK, Nakanishi Inc., Tokyo, Japan), at 350 rpm and 2.5 N. Each instrument was covered with RC Prep (Premier Dental Products Co, Plymouth Meeting, PA, USA) as a lubricant. The canals were irrigated with 3 mL of 2.5% sodium hypochlorite after each instrument. Each file series was used in one canal. Canal preparation was carried out by one operator. After the canal preparation, the specimens were once again placed in the same position, scanned and canal wall thicknesses measured. Data were statistically analyzed with ANOVA and post hoc test. The level of significant was set at 0.05.

## Results

The mean percentage changes in dentine thickness, standard deviation value and statistical analysis results are presented in Tables 1 and 2. The results showed that GG drills with hand system removed significantly more dentine than the rotary systems from different canal walls; (*P*<0.001). There were no significant differences between OneShape and ProTaper systems (*P*>0.05). Two-way ANOVA showed no statistically significant differences in dentine removal between 3 cross-section in each group (*P*>0.05).

## Discussion

The aim of this study was to compare the dentine thickness after root canal preparation with GG drills and hand system, ProTaper and OneShape rotary systems. The residual dentine thickness after root canal therapy is very important because excessive dentine removal increases the fracture of roots [20, 21]. The results showed more dentine removal in GG and hand instrumentation groups. However, there were no statistically significant differences between the two rotary file systems.

Lammertyn *et al. *[29] reported that dentine thickness depended on the furcal groove. In the cervical third, while the depth of groove increased, the palatal dentine thickness decreased [29]. In this study, CBCT imaging technique was used to obtain images and NTT software was used to measure dentine thickness of all walls without destroying the teeth [34, 38]. With the NTT program, horizontal sections were assessed below the furcation of roots because this area exhibits the greatest decrease in dentine thickness during preparation [7, 10]. The results showed that GG drills with hand files removed significantly more dentine compared to the rotary file systems and the ProTaper system removed more dentine than the OneShape system. Stainless steel files and GG drills are more rigid than the Ni-Ti instruments; therefore, these instruments tend to remove more dentine from the root canal walls [39]. With regard to our study, the amount of dentine removal with GG drills and hand files were significantly higher than those removed by rotary systems, which might be explained by more rigidity in GG drills and stainless steel than the Ni-Ti instruments.

**Figure1:**
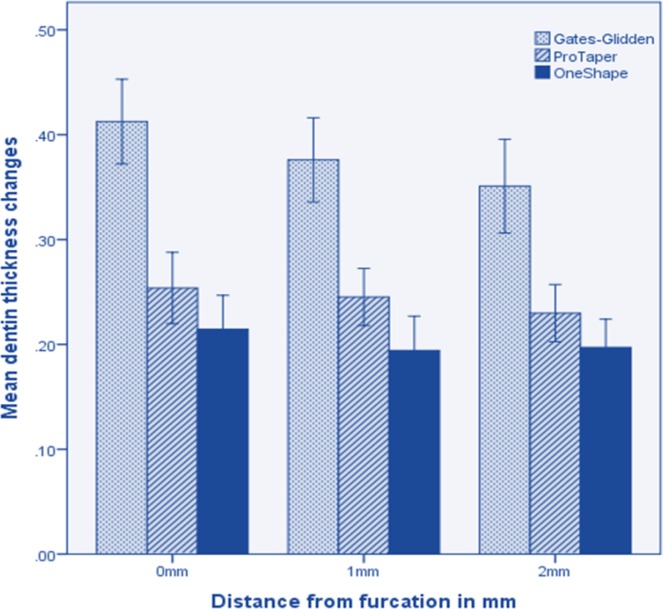
Mean changes of dentine thickness in the palatal aspect of buccal roots after preparation in all groups

Caution should be exercised when preparing canals with #3 GG drills. Zhang *et al. *[40] reported that the shaping files of ProTaper have an increasing taper from 3.5 % at D_0_ to 19% at D_9_ with higher elasticity; therefore these instruments prepare the coronal portion of the canals safety and without transportation [40]. The present study showed that OneShape rotary system removed less dentine from the all canal walls of root especially from the palatal walls of buccal roots. Figure 1 shows the mean changes of dentine thickness in the palatal aspect of buccal roots in all groups after preparation.

In addition, there were no statistically significant differences in 3 cross-sections between the instruments. The highest amount of dentine removal was detected in mesial walls in GG group, without significant difference, but it is an important point because the mesial walls of maxillary premolars is a critical area due to deep concavities on the cervical aspect [26, 29].

Mahran *et al. *[38] evaluated the effect of ProTaper, HeroShaper and GG drills with hand files on 3 mm bellow the orifice of mesiobuccal canal of first mandibular molars and reported that less dentine was removed with the use of ProTaper files on distal wall, compared to GG drills with hand files, but, the total dentine removed by ProTaper system was higher [38] presumably because of the last instrument used. Those researchers finished their preparation with F3 file, whereas the last file used in this study was F2.

**Table 1:** Mean (SD) of changes in coronal dentine thickness in buccal, lingual, mesial and distal walls in buccal root (different letters indicate statistical significance

	**Gates-Glidden**	**ProTaper**	**OneShape**	***Ρ*** **-value**
**Buccal Dentine**	31.79 ^a^ (22.4)	24.36 ^b^ (15.0)	20.77 ^b^ (11.5)	*Ρ*<0.001*
**Palatal Dentine**	32.56 (19.8)	23.08 (9.5)	18.46 (13.8)	*Ρ*<0.001*
**Mesial Dentine**	41.03 (19.5)	23.08 (18.2)	18.72 (18.0)	*Ρ*<0.001*
**Distal Dentine**	23.08 (18.0)	22.31 (12.6)	21.28 (19.6)	*Ρ*<0.001*

**Table 2:** Mean (SD) of changes in coronal dentine thickness in buccal, lingual, mesial and distal walls in palatal root (different letters indicate statistical significance

	**Gates-Glidden**	**ProTaper**	**OneShape**	***Ρ*** **-value**
**Buccal Dentine**	42.82 ^a^ (24.3)	32.31 ^b^ (14.5)	25.38 ^b^ (13.9)	*Ρ*<0.001*
**Palatal Dentine**	36.92 (24.1)	25.64 (20.3)	20.26 (16.3)	*Ρ*<0.001*
**Mesial Dentine**	44.10 (21.3)	21.79 (13.5)	20.77 (12.6)	*Ρ*<0.001*
**Distal Dentine**	36.15 (18.2)	21.79 (13.9)	15.90 (18.4)	*Ρ*<0.001*

Homayoon *et al. *[41] reported that there were no differences between different canal preparation systems (GG drills, ProTaper, K3 and RaCe) in relation to the amount of dentine removal in 1.5 mm cross-sections; however GG drills removed significantly more dentine compared to FlexMaster files 3 mm apical to the furcation [41]. In a study by Akhlaghi *et al. *[10], on comparison of the minimum residual root thickness, RaCe and ProTaper systems removed similar amounts of dentine, but #2,3 and 4 GG drills along with K-Flexofiles removed significantly more dentine compared to RaCe system and without significant difference from ProTaper [10]. Most studies showed no significant differences between GG drills and rotary system regarding dentine removal [7, 34, 42]. Duarte *et al. *[42] found that #20/0.06 LAAxxess instruments removed more dentine than #2 GG drills and #30/0.06 Orifice Shaper [42]

In a study by Sanfelice *et al. *[34] no differences were reported between GG drills, ProTaper, and K3 systems and LAAxxess instruments on cervical dentinee thickness, however in 2 recent studies [34, 42], they used only #1 and #2 GG drills while in this study #1, 2, and 3 instruments were used. 

Carvalho-Sousa *et al. *[7] did not report any significant differences in residual dentine after flaring with ProTaper rotary files and GG drills [7], however those researcher used different method for measuring dentine thicknesss.

In another study by Flores *et al. *[4] no significant differences were observed between GG, Largo, LAAxxess, and CPdrill on cervical dentinee thickness of mesiobuccal and mesiolingual canals of mandibular first molars [4].

Rolly *et al. *[13] reported that OneShape system indicated less transportation and canal preparation time compared to full subsequence ProTaper system [13]. Accordingly, root canal preparation with rotary file systems, especially OneShape file system for preparation of maxillary first premolars with seperate roots but additional studies are necessary for their cleaning and shaping abilities and trasortation potential.

## Conclusion

Based on the results of this study, GG drills with hand instruments removed significantly more dentine than rotary systems. Thus, it is recommended to prepare the canals of narrow and grooved roots with rotary instruments.
